# A Rare Case of Cavernous Hemangioma of the Mitral Valve Presenting As Multifocal Embolic Brain Infarcts

**DOI:** 10.7759/cureus.17721

**Published:** 2021-09-04

**Authors:** Om Parkash, Grace W Ying, Aatma Ram, Lalitha Padmanabha Vemireddy, Farah Zahra

**Affiliations:** 1 Internal Medicine, Chicago Medical School at Rosalind Franklin University of Medicine and Science, North Chicago, USA; 2 Internal Medicine, Chicago Medical School Internal Medicine Residency Program at Northwestern McHenry Hospital, McHenry, USA

**Keywords:** cavernous hemangioma, mitral valve, myxoma, fibroelastoma, infarcts

## Abstract

Primary cardiac tumors (PCTs) are rare and represent a heterogeneous group of tumors, potentially arising from various parts of the heart. The majority of these tumors are benign (90%), with myxoma being the most common subtype. Cardiac hemangiomas are rare vascular tumors that constitute 1-2% of all benign heart neoplasms. We present a rare case of a 79-year-old woman presented with multifocal embolic brain infarcts secondary to cavernous hemangioma of the mitral valve (MV). Cavernous hemangioma was successfully resected with follow-up imaging at six months, demonstrating no regrowth. There are no pathognomonic signs or findings to suggest cavernous hemangioma of the MV on clinical examination or imaging studies. Surgical resection and histopathologic analysis remain the gold standard for diagnosis and treatment, respectively. Following complete resection, the prognosis is generally favorable with a low recurrence rate, but periodic echocardiography is recommended to detect any potential recurrence.

## Introduction

Primary cardiac tumors (PCTs) are rare, with an incidence of between 0.0017% and 0.27% at autopsy, and in most cases are histologically benign [1]. Cardiac hemangiomas are rare vascular tumors representing 1-2% of all detected benign heart neoplasms [1]. Fewer than 50 cases of cardiac hemangiomas are reported in the literature. On further literature search, less than 15 mitral valve (MV) hemangiomas have been reported to date [1-10]. It is hard to differentiate hemangioma from other benign heart neoplasms clinically or through imaging studies. Their clinical manifestations depend on tumor location and size. Most common clinical manifestations include heart failure, arrhythmia, syncope, pseudo-angina, and thromboembolic events.

## Case presentation

**Figure 1:**
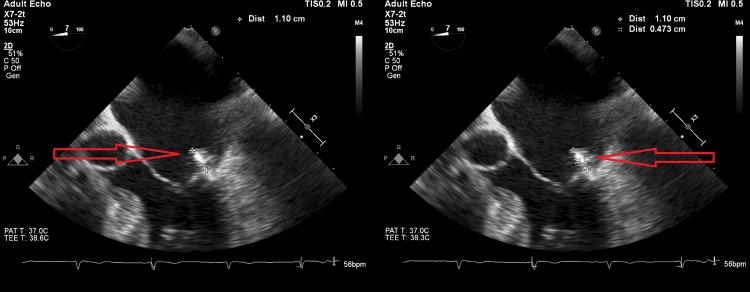
TEE demonstrating a pedunculated 1.0 × 0.7 cm mass on the annulus of the anterior leaflet of the MV, which appears to be connected by a stalk. MV: Mitral valve; TEE: Transesophageal echocardiogram.

**Figure 2:**
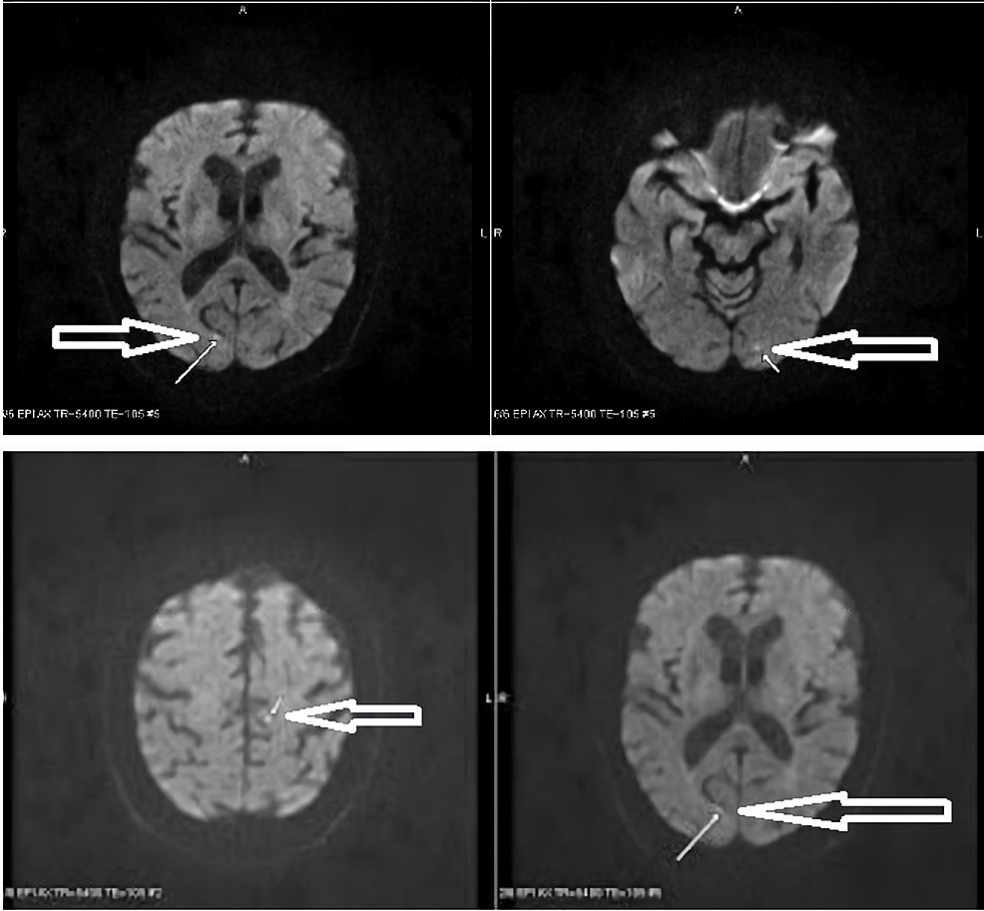
MRI brain demonstrating several punctate acute microinfarcts within the left frontal lobe and the bilateral occipital lobes.

A 78-year-old Caucasian woman presented with acute-onset dysarthria for 2-3 hours. Physical examination was unremarkable except for dysarthria. Vitals signs were normal. She had a past medical history of type 2 diabetes, coronary artery disease, chronic kidney disease, hypertension, and hyperlipidemia. She was admitted four months ago with symptoms of perioral numbness and underwent a full neurologic and cardiologic workup for stroke, which was negative for any significant findings. Subsequent transesophageal echocardiogram (TEE) revealed two MV pedunculated masses, clinically labeled as fibroelastomas. The patient was started on medical management with dual antiplatelet therapy (DAPT) instead of surgery. ECG obtained revealed normal sinus rhythm. The CT scan with and without contrast was negative for acute mass effect or bleeding. The TEE revealed two pedunculated masses that arise from the annulus by the medial side of P2. The larger mass measured 1.0 x 0.7 cm and the smaller one measured 0.5 x 0.5 cm (Figure 1). The MV masses were again suspected to be fibroelastomas with no changes since the last visit. The MRI of the brain found several punctate acute microinfarcts within the left frontal lobe and the bilateral occipital lobes without large territorial infarct, mild-moderate small vessel ischemic degenerative changes, and parenchymal volume loss (Figure 2). The magnetic resonance angiogram (MRA) of the brain and carotid arteries did not demonstrate any large vessel occlusion, flow-limiting stenosis, or aneurysm. The patient did not have any cardiac telemetry events during the hospital stay. The differential diagnoses after the initial workup included benign papillary fibroelastomas, myxomas, and vegetations secondary to infective endocarditis.

**Figure 3:**
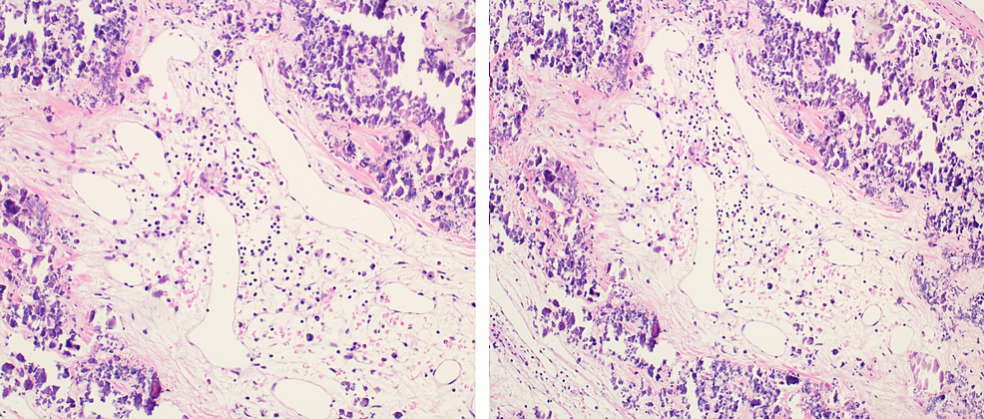
H&E stain showing thin-walled vascular spaces with dystrophic calcifications in adjacent connective tissue space.

The patient underwent open thoracotomy for resection of MV masses. During surgery, an inspection of the MV showed 1.5 x 0.5 cm and 0.5 x 0.5 cm calcified lesions along the annulus. These lesions were excised and sent for pathology. Interestingly, the histopathology examination reported it as a cavernous hemangioma (Figure 3). The patient was discharged home without any postoperative complications. Follow-up TEE in six months showed no regrowth of tumors.

## Discussion

PCTs are rare and often found incidentally on echocardiography or chest imaging. It is generally difficult to differentiate between different types of intracardiac mass on imaging. Common differentials include benign papillary fibroelastomas, myxomas, intracardiac thrombus, rhabdomyoma, fibromas, hemangiomas, teratomas, lipomas, cardiac hamartomas, intracardiac pheochromocytomas and paragangliomas [1, 2]. Cardiac hemangiomas are rare benign heart neoplasms. Demographically, hemangiomas have been detected in almost all age groups. However, there is a slight predominance among female patients. Their presentation depends upon the location, mobility, and size of the tumor. Important clinical implications include thromboembolic events, heart failure due to flow obstruction, suspicion of infective endocarditis, syncope, pseudo-angina, and arrhythmia [1, 2]. In some cases, they can also invade the lung and cause pulmonary symptoms [3].

Hemangiomas are vascular tumors composed of blood vessels lined by endothelial cells. Cardiac hemangiomas are sporadic vascular tumors representing 1-2% of all detected benign heart neoplasms [1, 4]. Cardiac hemangiomas can occur in any part of the heart. The most commonly involved sites are the epicardium and the myocardium. In addition, in terms of the anatomic sites of origin, slightly more than one-third (36%) of the cardiac hemangiomas are found in the right ventricle, another one-third (34%) are from the left ventricle. Approximately 23% of the cardiac hemangiomas are located in the right atrium (23%) and only a very few (7%) cases are found in the left atrium [5]. Valvular involvement of hemangioma is an extremely rare phenomenon due to the avascular anatomy of the heart valves (except for the peripheral one-third located near the valve ring) [1]. About 50 cases of cardiac hemangiomas have been reported in the literature. However, to the best of our knowledge and research, less than 15 cases of surgically treated cavernous hemangiomas of the MV have been reported in the medical literature so far [1-10].

Cardiac hemangiomas are classified as capillary, cavernous, and arteriovenous. Predominantly intramural hemangiomas are arteriovenous or cavernous, whereas endocardial hemangiomas are capillary. The gross appearance of hemangiomas generally depends on their location. Most intramural hemangiomas appear to be poorly circumscribed masses with a hemorrhagic or congested discoloration [4, 5]. Upon gross examination, they may appear as red-blue, soft, spongy masses. Microscopic examination findings of cavernous hemangiomas consist of large, endothelial lined, and blood-containing spaces separated by sparse connective tissue [5, 6]. Among the different types of cardiac hemangiomas, cavernous hemangiomas are associated with a slow growth rate and low invasion rate of the adjacent structures [5]. There are no pathognomonic signs and findings of cardiac hemangiomas that can differentiate these tumors, clinically or through imaging, from other intracardiac neoplasms. Transthoracic echocardiography and TEE have good sensitivity and accuracy in finding these neoplasms in the heart. Imaging modalities such as CT and MRI are very useful in diagnosing and evaluating the surgical resectability of the hemangioma [9, 10]. Coronary angiography often helps to establish the diagnosis; non-specific findings are vascular blush, which can also be found in cardiac myxomas [7, 8]. Histopathology and surgical resection remain the gold standard for diagnosis and treatment, respectively. Following complete resection, the prognosis is generally favorable with a low recurrence rate, but periodic echocardiography is performed to detect the potential recurrence of this tumor [1, 5].

## Conclusions

Cavernous hemangioma involving the MV is an infrequent phenomenon. We report a case of MV cavernous hemangioma presenting as a thromboembolic phenomenon causing multiple brain infarcts. Although cavernous hemangioma is a histopathological finding, it can present as thromboembolic events, heart failure due to obstruction, arrhythmia, pseudo-angina, or mimicking infective endocarditis. Surgical resection of the mass involving the MV is usually therapeutic. Recurrence of cavernous hemangioma has been reported in the medical literature. It is recommended to do periodic echocardiography to look for possible recurrence.
